# Distinct gene alterations between Fos‐expressing striatal and thalamic neurons after withdrawal from methamphetamine self‐administration

**DOI:** 10.1002/brb3.1378

**Published:** 2019-07-31

**Authors:** Xuan Li, Ian R. Davis, Olivia M. Lofaro, Jianjun Zhang, Raffaello Cimbro, F. Javier Rubio

**Affiliations:** ^1^ Department of Psychology University of Maryland College Park College Park Maryland; ^2^ Intramural Research Program NIDA, NIH, DHHS Baltimore Maryland; ^3^ CAS Key Laboratory of Mental Health Institute of Psychology Beijing China; ^4^ Division of Rheumatology, School of Medicine Johns Hopkins University Baltimore Maryland

**Keywords:** anterior intralaminar nucleus of thalamus, dorsal striatum, fluorescence‐activated cell sorting, Fos, incubation of drug craving, methamphetamine relapse

## Abstract

**Background:**

Methamphetamine (Meth) seeking progressively increases after withdrawal (incubation of Meth craving). We previously demonstrated a role of anterior intralaminar nucleus of thalamus (AIT) to dorsomedial striatum (DMS) projections in this incubation. Here, we examined molecular alterations in DMS and AIT neurons activated (identified by neuronal activity marker Fos) during “incubated” Meth‐seeking relapse test after prolonged withdrawal.

**Methods:**

We trained male rats to self‐administer Meth or saline (control condition) for 10 days (6 hr/day). Using fluorescence‐activated cell sorting, we examined gene expression in Fos‐positive (activated during a 2‐hr relapse test) and Fos‐negative (nonactivated) DMS and AIT neurons.

**Results:**

In DMS, we found increased mRNA expressions of immediate early genes (IEGs) (*Arc*,* Egr1*,* Npas4*,* Fosb*), *Trkb*, glutamate receptors subunits (*Gria3*,* Grin1*,* Grin2b*,* Grm1*), and epigenetic enzymes (*Hdac3*,* Hdac5*, *Crebbp*) in Fos‐positive neurons, compared with Fos‐negative neurons. In AIT, we found that fewer genes (*Egr1*, *Fosb*, *TrkB*, *Grin1*, *and Hdac5*) exhibited increased mRNA expression in Fos‐positive neurons. Unexpectedly, in both brain regions, gene alterations described above also occurred in drug‐naïve saline self‐administration control rats.

**Conclusions:**

These results demonstrated that transcriptional regulations in Fos‐positive neurons activated during the relapse tests are brain region‐specific but are not uniquely associated with drug exposure during the self‐administration training.

## 1 INTRODUCTION

A key challenge for treating methamphetamine (Meth) addiction is relapse during abstinence (Elkashef et al., 2008). In rats with a history of Meth self‐administration, Meth seeking progressively increases after prolonged withdrawal (Adhikary et al., 2017; Li, Zeric, Kambhampati, Bossert, & Shaham, 2015; Shepard, Bossert, Liu, & Shaham, 2004). This incubation phenomenon also occurs in Meth‐dependent patients during the first 3 months of abstinence (Wang et al., 2013). We and others previously identified several molecular mechanisms underlying this incubation, including critical roles of calcium‐permeable AMPA receptors in nucleus accumbens (Scheyer et al., 2016) and histone deacetylase 5 (HDAC5) in dorsal striatum (DS) (Li, Carreria, et al., 2018). Previous studies also used genome‐wide approaches and demonstrated distinct transcription profiles in striatum (Cadet, Brannock, Jayanthi, & Krasnova, 2015), central amygdala, and orbitofrontal cortex (Cates et al., 2018) during this incubation.

Recently, we explored the role of dorsomedial striatum (DMS and its afferent projections in incubation of Meth craving; Li, Rubio, et al., 2015; Li, Witonsky, et al., 2018). We found that DMS activation (assessed by the activity marker Fos, Cruz et al., 2013) is associated with this incubation and that DMS injections of SCH23390, a D1‐family receptor (D1R) antagonist that decreases striatal Fos expression (Valjent et al., 2000), decreased incubated Meth seeking after 30 withdrawal days (Li, Rubio, et al., 2015). We also used retrograde tracer (injected into DMS) in combination with Fos and found that activation of the anterior intralaminar nucleus of thalamus (AIT) and AIT‐to‐DMS projections is associated with incubated Meth seeking (Li, Witonsky, et al., 2018). Additionally, reversible inactivation of the lateral AIT (AIT‐L; but not medial AIT [AIT‐M]) and reversible asymmetric inactivation (Bossert et al., 2012) of AIT‐L‐to‐DMS glutamatergic projections and local D1R signaling in DMS decreased incubated Meth seeking (Li, Witonsky, et al., 2018). These studies demonstrate that activation of DMS, AIT‐L, and AIT‐L‐to‐DMS projections is critical to incubation of meth craving.

Based on the above findings, here we used fluorescence‐activated cell sorting (FACS) and examined mRNA expression of several immediate early genes (IEGs) and candidate genes in DMS and AIT neurons activated (Fos‐positive) during incubated Meth seeking. We examined AIT instead of AIT‐L because of technical limitations during tissue collection. Our choice for IEGs and candidate genes was based on our previous study where we demonstrated selective increases in mRNA expression of *Bdnf* and *Trkb*, several glutamate receptor subunits, and epigenetic enzymes in Fos‐positive dorsal striatal neurons activated during incubated Meth seeking (Li, Rubio, et al., 2015). We included two additional IEGs: *Fosb* (induced after acute noncontingent cocaine (Hope, Kosofsky, Hyman, & Nestler, 1992) and Meth exposure (Liu et al., 2014)), and *Npas4* (recently implicated in cocaine‐seeking behaviors (Taniguchi et al., 2017)). To determine whether the molecular alterations in Fos‐positive neurons are unique to incubated drug‐seeking behavior, we also included a drug‐naïve saline self‐administration control group that experienced the same behavioral procedures as the Meth self‐administration experimental group, except that lever presses led to saline infusions. This control condition was different from the drug‐experience no‐relapse‐test control group that was used in our previous FACS studies on relapse‐ and incubation‐associated molecular alterations in the Fos‐positive neurons (Fanous et al., 2013; Li, Rubio, et al., 2015; Rubio et al., 2015).

We hypothesized that DMS and AIT neurons activated during the relapse tests (Fos‐positive neurons) would exhibit distinct gene alterations compared with the surrounding Fos‐negative neurons and that these gene alterations will be uniquely associated with meth self‐administration, withdrawal, and drug relapse testing. However, we found similar gene alterations in Fos‐positive DMS and AIT neurons activated during the late withdrawal (day 30) relapse/incubation tests in the drug‐naïve saline self‐administration control rats. Therefore, to determine whether the gene expression pattern in DMS and AIT Fos‐positive neurons generally occurs in response to other Fos‐inducing stimuli, we examined mRNA expression of the same genes in DMS and AIT neurons activated by acute novel context exposure, a condition known to induce strong Fos expression in cortical and subcortical regions (Badiani et al., 1998; Uslaner et al., 2001).

## 2 METHODS AND MATERIALS

### 2.1 Subjects

We used male Sprague‐Dawley rats (Charles River, total *n* = 35), weighing 300–350 g prior to surgery and 325–375 g at the start of the drug self‐administration procedure; we maintained the rats under a reverse 12:12‐hr light/dark cycle with food and water freely available. We kept the rats two per cage prior to surgery and then housed them individually after surgery. We performed the experiments in accordance with the National Institutes of Health Guide for the Care and Use of Laboratory Animals (8th edition), under the protocols approved by the Animal Care and Use Committee. We excluded three rats, due to poor training or health‐related issues.

### 2.2 Intravenous surgery

We anesthetized the rats with isoflurane gas (5% induction; 2%–3% maintenance) and inserted silastic catheters into the rat's jugular vein, as previously described (Caprioli et al., 2017; Li, Carreria, et al., 2018). We injected the rats with ketoprofen (2.5 mg/kg, s.c.) after surgery to relieve pain and inflammation; we allowed the rats to recover 5–7 days before saline or Meth self‐administration training. During the recovery and training phases, we flushed the catheters every 24–48 hr with gentamicin (Butler Schein; 5 mg/ml) dissolved in sterile saline.

### 2.3 Apparatus

We trained the rats in self‐administration chambers located inside sound‐attenuating cabinets and controlled by a Med Associates (Georgia, VT) system. Each chamber has two levers located 8–9 cm above the floor. During self‐administration training, presses on the retractable (active) lever activated the infusion pump (which delivered a Meth or saline infusion); presses on the stationary (inactive) lever were not reinforced. For intravenous infusions, we connected each rat's catheter to a liquid swivel (Instech) via polyethylene‐50 tubing, protected by a metal spring. We then attached the liquid swivel to a 20‐ml syringe via polyethylene‐50 tubing and to a 22‐gauge modified cannula (Plastics One).

### 2.4 Meth and saline self‐administration training

We used an extended access training procedure based on our previous study (Li, Witonsky, et al., 2018). We trained the rats to self‐administer Meth or saline (as the control condition) for 6 hr per day under a fixed‐ratio‐1 (FR‐1) 20‐s time‐out reinforcement schedule. We dissolved Meth in saline, and the rats self‐administered Meth at a dose of 0.1 mg/kg/infusion over 3.5 s (0.1 ml/infusion). We trained the rats for 10 sessions over a 11‐day period (one off‐day after the 4th or 5th training day) to prevent loss of body weight during the training phase. (Note: Meth‐trained rats lost about 10–15 g after 4 or 5 days of training and regained the lost weight during the off‐day [data not shown]).

**Figure 1:**
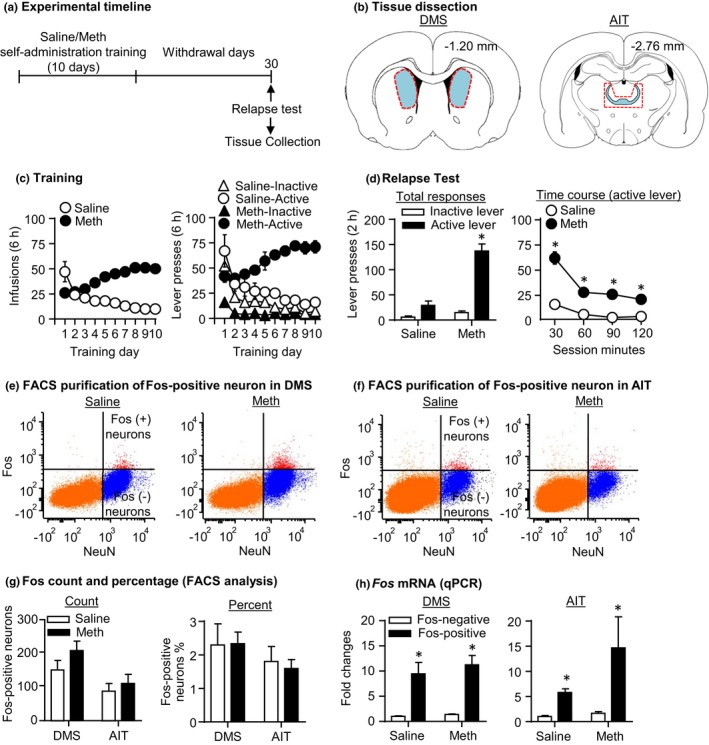
FACS of Fos‐expressing neurons activated during relapse tests after prolonged withdrawal from saline and Meth self‐administration (Exp. 1). (a) Timeline of the experiment. (b) Representative schematics of DMS and AIT tissue collection (blue areas represent regions of interest; red dotted lines represent areas collected during free‐hand dissection; Paxinos & Watson, 2005). (c) Saline and Meth self‐administration. Data are mean ± *SEM* number of saline of Meth (0.1 mg/kg/infusion) infusions or lever presses during the ten 6‐hr daily self‐administration sessions for Exp. 1 (total *n* = 24). During training, active lever presses were reinforced on an FR1 20‐s time‐out reinforcement schedule, and saline or Meth infusions were paired with a 5‐s tone–light cue. (d) Relapse test on withdrawal day 30: Data are mean ± *SEM* of responses on previously active lever and on the inactive lever during the 2‐hr relapse test on withdrawal day 30. During testing, lever presses led to contingent presentations of the tone–light cue paired previously with saline/Meth infusions during training. *Different from saline, *p* < .05, *n* = 10–14 per group. (e) and (f) Examples of FACS of NeuN‐positive (*x*‐axis) and Fos‐positive or Fos‐negative (*y*‐axis) cells (Fos‐positive and Fos‐negative neurons) in DMS and AIT of saline and Meth animals, respectively. Fos‐positive and Fos‐negative neurons are located in the top right and bottom right quadrants, respectively. (g) Sorted Fos‐positive neurons by FACS. Left, total number. Right, percentage of total NeuN‐positive neurons. (h) Fos mRNA expression of sorted Fos‐positive neurons. Data are presented as folds of mean values in Fos‐negative neurons from the saline group. *Different from Fos‐negative neurons, *p* < .05, *n* = 10–14 per group. Error bars indicate *SEM*

The daily training sessions started at the onset of the dark cycle. The sessions began with the extension of the active lever and the illumination of the red house light, which remained on for the duration of each session. During training, active lever presses led to the delivery of a Meth or saline infusion and a compound 5‐s tone–light cue (the tone and light modules [Med Associates] were located above the active lever). During the 20‐s time‐out, we recorded nonreinforced active lever presses. To prevent overdose, we set the maximal number of daily drug intake to less than 90 infusions. The red house light was turned off and the active lever retracted after the rats received the maximal infusions or at the end of each session. The experimental conditions for the saline group were identical to those of the Meth group, except that lever presses resulted in the delivery of saline infusions. The training data are described in Figure 1c.

### 2.5 Withdrawal phase

During this phase, we housed the rats individually in the animal facility and handled them 3–4 times per week.

### 2.6 Extinction (relapse) tests

We conducted the relapse test immediately after the onset of the dark cycle. The sessions began with the extension of the active lever and the illumination of the red house light, which remained on for the duration of the session. Active lever presses during testing (the operational measure of drug seeking in incubation of craving and relapse/reinstatement studies, Pickens et al., 2011; Reiner, Fredriksson, Lofaro, Bossert, & Shaham, 2019; Shalev, Grimm, & Shaham, 2002; Venniro, Caprioli, & Shaham, 2016) resulted in contingent presentations of the tone–light cue, previously paired with Meth infusions, but not the drug itself (i.e., under extinction conditions). The saline group underwent the same test as the Meth group.

### 2.7 Novel context exposure

We exposed the rats to a novel environment by placing them in a large plastic round bowl with new beddings in a different room from their home‐cage holding room.

### 2.8 FACS

We performed live decapitation and obtained a 2‐mm coronal section containing the striatum (between approximately Bregma anteroposterior + 2.28 and + 0.36 mm) and thalamus (between approximately Bregma anteroposterior −1.80 and −3.60 mm; Paxinos & Watson, 2005) using a brain matrix (ASI Instruments). We dissected DMS and AIT with razor blades, as shown in Figure 1b. We froze the brain tissue in microcentrifuge tubes on dry ice and stored tissue at −80°C.

We processed the frozen tissue for FACS as described in Ref. (Rubio, Li, Liu, Cimbro, & Hope, 2016). Briefly, we retrieved the frozen tissue with fine tweezers and placed the tissue on the cold glass plate on wet ice. We added 1–2 drops of ice‐cold Hibernate A (HA‐if, Brain Bits) to cover the tissue. We allowed the tissue to thaw for 2 min before mincing. We finely minced the tissue with razor blades and transferred the tissue into 1 ml of Hibernate A. After centrifuging the tissue for 2 min at 110 *g* (4°C), we added 1 ml of Accutase (SCR005; Millipore) and mixed up and down 4 times prior to digesting the tissue for 30 min at 4°C with end‐over‐end mixing. We then centrifuged the tissue for 2 min at 960 *g* (4°C) and resuspended the pellet in 0.6 ml of ice‐cold Hibernate A.

We first triturated each tissue sample three times in series using fire‐polished glass pipettes with successively smaller diameters (1.3, 0.8, and 0.4 mm) and then conducted three additional trituration steps with 0.4‐mm‐diameter glass pipettes. Each trituration step consisted of triturating up and down 10 times followed by 2 min on ice to sediment the larger debris and undissociated cells. We combined supernatant from each trituration step to obtain dissociated cells in a total volume of 3.6 ml. We then fixed and permeabilized cells by adding the same volume of 100% of cold ethanol (−20°C) for 15 min on ice. After collecting the cells by centrifugation (1,700 *g*, 4 min, 4°C), we resuspended the cells with 0.7 ml of cold PBS and then filtered the cells with 100‐µm and 40‐µm cell strainers (Falcon brand; BD Biosciences).

We incubated cells with PE‐labeled anti‐NeuN antibody (1:500 in PBS; FCMAB317PE; Millipore, RRID: AB_10807694) and Alexa 647‐labeled anti‐phospho‐c‐Fos antibodies (1:500 in PBS; 8677, Cell Signaling, RRID: AB_1178518) for 30 min at 4°C and then washed the cells with 0.8 ml cold PBS. After collecting the cells by centrifugation (1,300 *g*, 3 min, 4°C), we washed the cells again with 1 ml cold PBS, followed by centrifugation (1,300 *g*, 3 min, 4°C), and resuspended the cells in 0.5 ml cold PBS for sorting in a FACSAria I cell sorting (BD Biosciences).

As previously reported (Li, Rubio, et al., 2015; Liu et al., 2014), neurons can be identified based on the distinct forward (FSC) and side (SSC) scatter property. DAPI (1 µg/ml, DNA staining) staining showed that ~98% of the events in the neuron gate are DAPI‐positive events (nucleated cells). After defining the cell population, we gated single cells by FSC width and height (~98% of single‐cell populations were DAPI‐positive) and conducted subsequent sorting within this single‐cell population. We sorted neurons according to PE (NeuN‐immunopositive) and Alexa Fluor 647 (Fos‐immunopositive) fluorescence signal. We set the threshold of Alexa Fluor 647 fluorescence signal based on background fluorescence signals of Home‐cage‐naïve rats. We collected all NeuN‐positive + Fos‐positive events (Fos‐positive neurons) and a maximum of 5,000 NeuN‐positive + Fos‐negative events (Fos‐negative neurons). We analyzed the data using FCS Express 6 (De Novo Software).

### 2.9 RNA extraction, cDNA synthesis, and qPCR

We collected sorted cells directly into 50 µl of the extraction buffer from PicoPure RNA isolation kit (Arcturus Bioscience) and lysed the cells by pipetting up and down 10 times followed by incubation for 30 min at 42°C. After centrifuging the suspension at 3,000 *g* at 4°C for 2 min, we collected the supernatant for RNA extraction. We extracted RNA according to PicoPure RNA isolation protocol and synthesized single‐strand cDNA using the Superscript III first‐strand cDNA synthesis kit (Invitrogen) according to the manufacturer's protocol.

We used gene‐targeted pre‐amplification of cDNA as previously described (Li, Rubio, et al., 2015; Liu et al., 2014). Briefly, we used a pooled primer solution of 0.2× concentration of TaqMan ABI primer/probes (20× TaqMan Gene Expression Assay as the stocking solution) and 80 nM of customized primer sets (Table S1). Each cDNA sample (7.5 µl) was mixed with the pooled primer solution (7.5 µl) and 15 µl of 2× TaqMan PreAmp Master Mix (Applied Biosystems). We pre‐amplified cDNA in an ABI 9700 Thermal Cycler using the following program: 95°C hold for 10 min; denaturation at 90°C for 15 s; and annealing and extension at 60°C for 4 min (14 cycles). We diluted the pre‐amplified cDNA product 7 times before proceeding to qPCR. We performed qPCR in duplicates with a Fam‐labeled probe for each target gene and a Vic‐labeled probe for the endogenous control gene (NeuN). We used TaqMan^®^ Advanced Fast PCR Master Mix (Life Technologies) in 7500 Fast TaqMan instrument, using the following program: 95°C hold for 20 s; then 40 cycles with denaturation at 95°C for 3 s, and annealing and extension at 60°C for 30 s. We analyzed the reactions using the ΔΔCt method with NeuN as the housekeeping gene.

We chose *NeuN* as the housekeeping gene, to be consistent with our previous study (Li, Rubio, et al., 2015). We also analyzed *NeuN* Ct value by using the geometric means of the Ct values among *Gapdh*, *Pde10a* (phosphodiesterase 10a), and *β‐actin*, and found that there were no differences in *NeuN* expression across different experimental conditions (data not shown). We also verified the uniformity of the pre‐amplification step by comparing cDNA templates from the unamplified and pre‐amplified samples. All ΔΔCt values of the tested genes between pre‐amplified and unamplified cDNA samples were within the range of ±1.5 for all target genes between the pre‐amplified and unamplified samples (data not shown).

### 2.10 Exp. 1: mRNA expression of IEGs and candidate genes in DMS and AIT Fos‐expressing neurons after prolonged withdrawal from saline or Meth self‐administration

**Figure 2:**
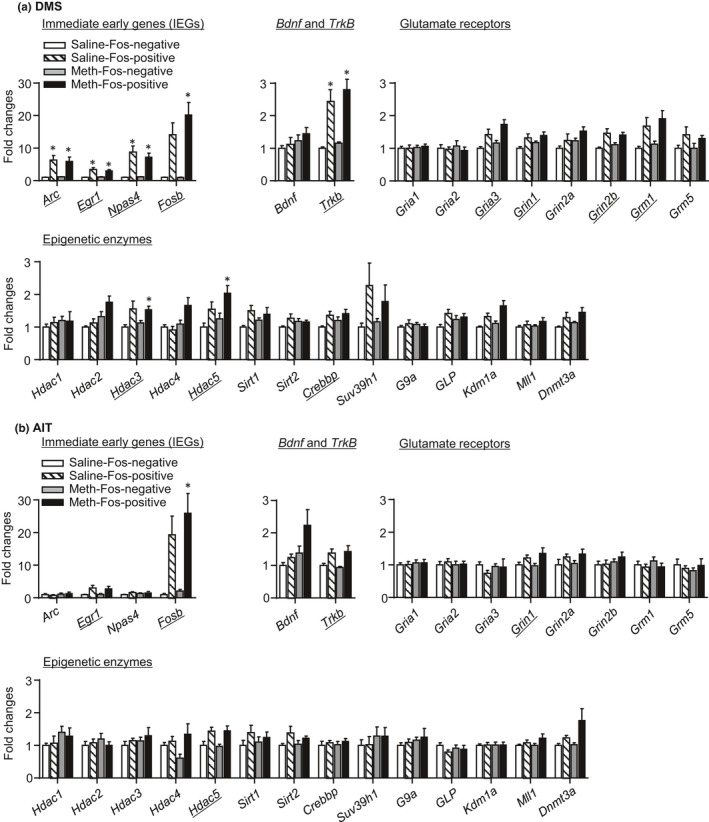
Immediate early gene, Bdnf and TrkB, glutamate receptor, and epigenetic enzyme mRNA expression in Fos‐positive neurons after prolonged withdrawal from saline and Meth self‐administration (Exp. 1). For both DMS (a) and AIT (b), data are presented as folds of mean values in Fos‐negative neurons from the saline group. Underlines indicate the significant main effect of Fos labeling. *Different from saline‐ or Meth‐ Fos‐negative neurons, respectively; *p* < .005, *n* = 5–14 per group. Error bars indicate *SEM*

We performed intravenous surgery on two groups of rats (saline: *n* = 10; Meth: *n* = 14) and trained them to self‐administer saline or Meth as described above. On withdrawal day 30, we tested both groups for extinction responding (2‐hr test). At the end of the relapse tests, we collected DMS and AIT tissue from both saline and Meth groups as shown in Figure 1b and froze them immediately on dry ice. We stored tissue at −80°C. Subsequently, we processed the tissue for FACS as described above. Next, we performed RNA extraction, cDNA synthesis, gene‐targeted pre‐amplification, and qPCR on FACS‐isolated neurons (see above). In FACS‐isolated Fos‐positive and Fos‐negative neurons, we measured mRNA expression of several IEGs, *Bndf* and *Trkb*, glutamate receptor subunits, and epigenetic enzymes (Figure 2).

### 2.11 Exp. 2: mRNA expression of IEGs and candidate genes in DMS and AIT Fos‐expressing neurons after acute exposure to novel context

As described in the Results section, the main finding in Exp. 1 was that there were similar changes in mRNA expression of IEGs and candidate genes in Fos‐positive neurons, activated during the extinction (relapse) tests, in DMS and AIT of rats with a history of either saline or Meth self‐administration (compared with the Fos‐negative neurons, respectively). In Exp. 2, we examined mRNA expression of IEGs and candidate genes in Fos‐positive neurons in DMS and AIT of rats after acute exposure to novel context to determine whether the gene expression changes reflect exposure to any non‐home‐cage environment.

We placed rats (*n* = 8) in a novel context environment for 2 hr. After the exposure, we collected DMS and AIT tissue, and frozen them immediately on dry ice. We stored tissue at −80°C. Subsequently, we processed the tissue for FACS as described above. Next, we performed RNA extraction, cDNA synthesis, gene‐targeted pre‐amplification, and qPCR on FACS‐isolated neurons (see above). In FACS‐isolated Fos‐positive and Fos‐negative neurons, we measured mRNA expression of the same genes as in Exp. 1 (Figure 4).

### 2.12 Statistical analysis

We analyzed the behavioral and molecular data with SPSS (version 24) or Prism GraphPad (version 7) using mixed ANOVAs, ANCOVAs, one‐way ANOVA, or *t* test, as appropriate. We followed significant interaction or main effects with Fisher PLSD post hoc tests. For the repeated measures analyses of the training data, we replaced seven outlier values of inactive lever presses with the group mean for a given training day. We defined outliers as three median absolute deviations (MADs) above the group median (Leys, Ley, Klein, Bernard, & Licata, 2013), and we only replace one outlier (the highest value above the threshold) for each training day. We indicate the between‐ and within‐subject factors of the different analyses in the Results section. To account for the multiple statistical comparisons of the mRNA expression data of the 27 genes in Figures 2 and 4, we set a conservative and more stringent significance criterion for these data (*p* < .005). We also excluded one outlier value above and/or below the threshold (defined by above or below three MADs from the group median), and samples with undetectable Ct values from the data presentation and statistical analysis of the molecular data. All statistical comparisons are listed in Tables S2 and S3.

## 3 RESULTS

### 3.1 Exp. 1: mRNA expression of IEGs and candidate genes in DMS and AIT Fos‐expressing neurons after prolonged withdrawal from saline or Meth self‐administration

The goal of Exp. 1 was to characterize the gene expression profile of DMS and AIT Fos‐positive neurons activated during 2‐hr extinction (relapse) tests after 30‐day withdrawal from saline or Meth self‐administration. For this purpose, we used FACS and obtained Fos‐positive and Fos‐negative neurons in DMS and AIT, respectively. We also measured mRNA expression of several IEGs, *Bdnf,* and *Trkb*, glutamate receptors, and epigenetic enzymes in both Fos‐positive and Fos‐negative neurons.

#### 3.1.1 Saline and Meth self‐administration (Figure 1c)

The Meth‐trained rats increased their drug intake across sessions, whereas the self‐administration behavior of saline rats decreased over time. We analyzed the infusion data with the between‐subject factor of Drug (saline, Meth) and the within‐subject factor of Session (1–10). We observed a significant interaction between the two factors (*F*_3,72_ = 37.25, *p* < .001, Figure 1c‐Left, Table S2). Analysis of the lever‐pressing data showed a significant triple interaction between the between‐subject factor of Drug (saline, Meth), and the within‐subject factors of Session (1–10) and Lever (active lever, inactive lever) (*F*_3,79_ = 8.79, *p* < .001, Figure 1c‐Right, Table S2).

#### 3.1.2 Extinction (relapse) tests (Figure 1d)

Extinction responding in the Meth‐trained rats was higher than in the saline‐trained rats after 30 withdrawal days. We analyzed the data with the between‐subject factor of Drug (saline, Meth) and the within‐subject factor of Lever (active lever, inactive lever). We observed a significant interaction between the two factors (*F*_1,22_ = 36.49, *p* < .001, Figure 1d‐Left, Table S2). Analysis of the time course (30‐min interval) of the active lever presses showed a significant interaction between Drug and Session minutes (*F*_1.7,36.9_ = 12.71, *p* < .001, Figure 1d‐Right, Table S2).

#### 3.1.3 FACS of Fos‐positive neurons (Figure 1e–h)

The total numbers and percentages of Fos‐positive neurons were similar between saline and Meth rats in DMS and AIT, respectively (Figure 1e–g). Additionally, we found that *Fos* mRNA expression in Fos‐positive neurons significantly increased compared with its respective Fos‐negative neurons, which validated the cell type of sorted Fos‐positive neurons. We analyzed *Fos* mRNA expressions in DMS or AIT with the between‐subject factor of Drug (saline, Meth) and the within‐subject factor of Fos labeling (negative, positive). We observed no interaction between Drug and Fos labeling, but a main effect of Fos labeling (DMS: *F*_1,19_ = 40.85; AIT: *F*_1,19_ = 6.56 *p* < .001, Figure 1h, Table S2).

#### 3.1.4 IEG and candidate gene expression in Fos‐positive neurons (Figure 2)

In DMS (Figure 2a), mRNA expression of IEGs and several candidate genes increased in Fos‐positive neurons compared with Fos‐negative neurons in both saline and Meth rats. In AIT (Figure 2b), the expression of fewer IEGs and candidate genes increased in Fos‐positive neurons compared with DMS. We analyzed mRNA expressions with the between‐subject factor of Drug (saline, Meth) and the within‐subject factor of Fos labeling (negative, positive). For DMS, we observed no interactions between Drug and Fos labeling for all genes (Table S2), but main effects of Fos labeling for IEGs (*Arc*, *Egr1*, *Npas4*, and *Fosb*), *Trkb*, glutamate receptor subunits (*Gria3*, *Grin1*, *Grin2b,* and *Grm1*), and epigenetic enzymes (*Hdac3*, *Hdac5,* and *Crebbp*) (*p* < .005, Figure 2a and Table S2). For AIT, we observed no interactions between Drug and Fos labeling for all genes (Table S2), but main effects of Fos labeling for *Egr1*, *Fosb*, *Trkb*, *Grin1,* and *Hdac5* (*p* < .005, Figure 2b and Table S2).

In summary, the data in Exp. 1 demonstrate that DMS or AIT neurons activated during the relapse tests exhibited distinct gene expression profiles. However, similar gene alterations occurred in Meth‐experienced rats and the drug‐naïve saline‐experienced rats in DMS and AIT, respectively.

### 3.2 Exp. 2: mRNA expression of IEGs and candidate genes in DMS and AIT Fos‐expressing neurons after acute exposure to novel context

To determine whether the molecular changes in the Fos‐positive neurons observed in Exp. 1 simply reflect differences between stimulus‐induced activated versus nonactivated neurons independent of the past history of the rats (e.g., self‐administration and relapse tests), we examined mRNA expression of the same genes as in Exp. 1 in DMS and AIT neurons activated by novel context exposure, a condition known to strongly increase IEGs expression in the brain (Badiani et al., 1998; Uslaner et al., 2001).

#### 3.2.1 FACS of Fos‐positive neurons (Figure 3b–d)

**Figure 3:**
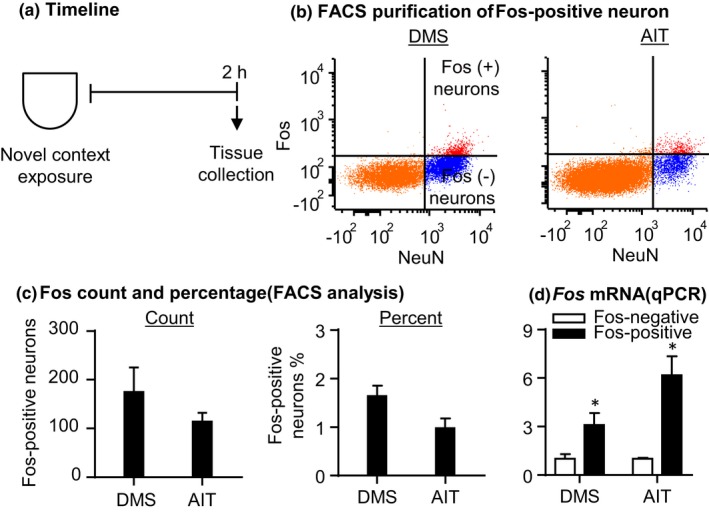
FACS of Fos‐expressing neurons activated during exposure to novel context (Exp. 2). (a) Timeline of the experiment. (b) Examples of FACS of NeuN‐positive (*x*‐axis) and Fos‐positive or Fos‐negative (*y*‐axis) cells (Fos‐positive and Fos‐negative neurons) in DMS and AIT. Fos‐positive and Fos‐negative neurons are located in the top right and bottom right quadrants, respectively. (c) Sorted Fos‐positive neurons by FACS. Left, total number. Right, percentage of total NeuN‐positive neurons. (h) Fos mRNA expression of sorted Fos‐positive neurons. Data are presented as folds of mean values in Fos‐negative neurons. *Different from Fos‐negative neurons, *p* < .05, *n* = 5–6 per group. Error bars indicate *SEM*

The total number and percentage of Fos‐positive neurons in DMS and AIT (Figure 3c) were comparable to the values in Exp. 1 (Figure 1g). Additionally, we analyzed *Fos* mRNA expression with paired *t* tests and found that *Fos* mRNA expressions in Fos‐positive neurons significantly increased compared with its respective Fos‐negative neurons (Figure 3d), which validated the cell type of sorted Fos‐positive neurons.

#### 3.2.2 IEG and candidate gene expression in Fos‐positive neurons (Figure 4)

**Figure 4:**
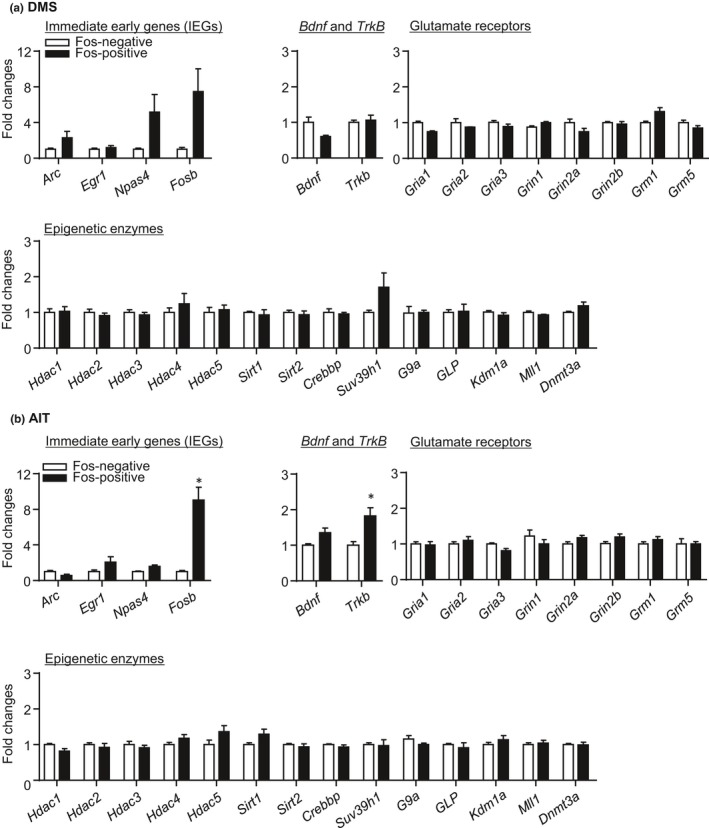
Immediate early gene, Bdnf and Trkb, glutamate receptor, and epigenetic enzyme mRNA expression in Fos‐positive neurons activated during acute exposure to novel context (Exp. 2). For both DMS (a) and AIT (b), data are presented as folds of mean values in Fos‐negative neurons. *Different from Fos‐negative neurons, *p < .005, *n* = 4–8 per group. Error bars indicate *SEM*

We observed minimal changes in IEG and candidate gene expressions in Fos‐positive neurons compared with Fos‐negative neurons after acute exposure to the novel context. We used paired *t* tests and analyzed mRNA expressions in Fos‐positive and Fos‐negative neurons. In DMS, we found no significant changes in IEG and candidate gene expressions in Fos‐positive neurons, compared with Fos‐negative neurons (Figure 4a, Table S2). In AIT, we observed significant increases in *Fosb* (*t*_6_ = 5.24, *p* = .002) and *Trkb* (*t*_6_ = 4.99, *p* = .003) mRNA expression, but no changes in other IEGs and candidate genes in Fos‐positive neurons, compared with Fos‐negative neurons (Figure 4b, Table S2).

In summary, the data in Exp. 2 demonstrate that DMS and AIT neurons activated by novel context exposure exhibited distinct gene expression profiles. Furthermore, the changes in both IEGs and candidate gene expression were minimal compared with Exp. 1.

## 4 DISCUSSION

We used FACS and examined mRNA expression of IEGs and candidate genes in DMS and AIT neurons activated either during the relapse tests after prolonged withdrawal from saline and Meth self‐administration (Exp. 1), or activated by acute exposure to novel context (Exp. 2). Our main finding is that DMS and AIT exhibited distinct molecular profiles of Fos‐positive neurons activated by the relapse tests after Meth self‐administration. However, contrary to our hypothesis in both brain regions, alterations of mRNA expression of IEGs, *Trkb,* glutamate receptors, and epigenetic enzymes were similar in rats with a history of saline versus Meth self‐administration. In contrast, we observed minimal gene alterations of Fos‐positive neurons in rats exposed to novel context. Together, these findings suggest that transcriptional regulation in Fos‐positive neurons is region‐specific, but unexpectedly is not uniquely associated with Meth exposure during the self‐administration training or exposure to Meth‐associated contexts and cues during the relapse tests.

### 4.1 Direct comparison of molecular alterations in Fos‐positive neurons between DMS and AIT

Our data provided a direct comparison of molecular alterations of Fos‐positive activated neurons between DMS and AIT, both associated with incubated Meth seeking (Li, Rubio, et al., 2015; Li, Witonsky, et al., 2018). This comparison is novel because no FACS studies have previously examined Fos‐positive neurons in more than one behaviorally relevant brain region in the same behavioral procedure (Fanous et al., 2013; Guez‐Barber et al., 2011; Li, Rubio, et al., 2015; Liu et al., 2014; Rubio et al., 2015).

In the self‐administration study (Exp. 1), we observed selective increases in other IEGs (*Arc*, *Egr1*, *Npas4*, and *Fosb*), glutamate receptors (*Gria3*,* Grin1*,* Grin2b*,* Grm1*), and epigenetic enzymes (*Hdac3*,* Hdac5*, *Crebbp*) in DMS neurons activated during the relapse tests, which extends previous findings in the whole dorsal striatal neurons activated by context‐induced Meth seeking (Rubio et al., 2015) and incubated Meth seeking (Li, Rubio, et al., 2015). Induction of *Npas4* expression also extends a recent study examining the role of striatal *Npas4* in cocaine relapse (Taniguchi et al., 2017). It is of note that mRNA expression of several genes (e.g., *Mll1* and *Hdac5*) in Fos‐positive neurons here was inconsistent to our previous study (Li, Rubio, et al., 2015). These discrepancies may be due to the fact that our previous study focuses on the whole dorsal striatum (Li, Rubio, et al., 2015), while the current study examined DMS, a subregion of dorsal striatum. In addition, our previous study (Li, Rubio, et al., 2015) also examined the homogenate of the whole dorsal striatum, which includes both neurons and glial cells. Therefore, direct comparison between these studies should be made with caution.

In AIT, we observed overall fewer molecular alterations, including IEGs and candidate genes in Fos‐positive neurons activated during the relapse tests than in the DMS. For example, we observed no induction of *Arc* and *Npas4* expression in Fos‐positive AIT neurons activated during the relapse tests, which suggest that neither *Arc* nor *Npas4* is a sensitive neuronal activity marker in AIT. An alternative explanation is that *Arc* or *Npas4* expression was induced in a group of activated neurons distinct from Fos‐positive neurons in AIT. For example, a previous study shows that after acute methamphetamine exposure, 20 percent of dorsal striatal Arc‐positive neurons do not express Fos (Liu et al., 2014).

In the novel context study (Exp. 2), we observed no significant changes in IEGs and other candidate genes in DMS Fos‐positive neurons. These data suggest that the transcriptional regulations in DMS Fos‐positive neurons observed in Exp. 1 are specific to shared experiences in the meth‐ and saline‐exposed rats in this experiment. In AIT, we observed *Fosb* and *Trkb*, upregulated in Fos‐positive neurons in Exp. 1, also increased in Fos‐positive neurons activated by the novel context. These data suggest that transcriptional regulation of *Fosb* and *Trkb* in AIT Fos‐positive neurons reflects the general role of AIT in arousal and awareness (Groenewegen & Berendse, 1994; Pereira de Vasconcelos & Cassel, 2015; Schiff, 2008; Van der Werf, Witter, & Groenewegen, 2002).

### 4.2 Molecular alterations in Fos‐positive neurons activated during drug seeking

An unexpected finding in our study was that regardless of Meth or saline experience during self‐administration training, DMS and AIT neurons activated during the relapse tests exhibited similar increases in IEGs and other candidate genes. This unexpected finding is against our original hypothesis that molecular alterations in Fos‐positive neurons activated are unique to drug‐seeking behavior. We speculate that these similarities were due to shared previous history of surgery, exposure to the self‐administration chambers, daily transfer and handling, and re‐exposure to the self‐administration context. Our speculations are consistent with the observation that in rats with novel context exposure only, the gene alterations were minimal compared with the rats in Exp. 1.

Two major limitations should be taken into consideration when interpreting these data. First, we cannot determine whether neurons activated in saline or Meth rats are the same group of neurons or not. Based on a recent study showing that distinct Fos‐expressing neurons mediate food reward and extinction memory, respectively (Warren et al., 2016), it is likely that the Fos‐positive neurons in saline‐ and Meth‐experienced rats comprise of different groups of neurons. Moreover, these Fos‐positive neurons may also belong to different cell types such as neurons expressing dopamine 1 versus dopamine 2 receptor. Therefore, direct comparison between these Fos‐positive neurons in saline and meth rats should be made with caution. Another limitation is that with the current tools available, we have not yet been able to identify activated neurons before they become activated (e.g., prior to the relapse test). Therefore, we cannot differentiate genes rapidly induced during the relapse tests or gradually induced after withdrawal from Meth in the current FACS study.

Our results question the uniqueness of the molecular alterations (to drug seeking) observed in the behaviorally activated cortical and striatal neurons in previous incubation studies (Fanous et al., 2013; Li, Rubio, et al., 2015) and underscore the importance of incorporating nondrug control groups in molecular studies of behaviorally activated neurons in future studies. However, our data do not refute the hypothesis that certain molecular alterations in the Fos‐positive neurons are specific to drug seeking. A comprehensive examination of this hypothesis can only be done using genome‐wide transcriptional analyses (e.g., RNA sequencing), which is beyond the scope of our study. Indeed, two previous studies also provided supporting evidence for this hypothesis. First, in our previous study where we used the context‐induced reinstatement of drug‐seeking model (Crombag, Bossert, Koya, & Shaham, 2008), we found unique molecular alterations in striatal Fos‐expressing neurons activated by exposure to the drug‐paired context, but not the extinction‐paired context (Rubio et al., 2015). Second, a recent electrophysiological study demonstrated greater firing capacities of Fos‐positive striatal neurons (labeled by GFP in FosGFP mice, compared with the surrounding Fos‐negative neurons) activated by a context previously paired with noncontingent cocaine exposure, but not saline exposure (Ziminski, Sieburg, Margetts‐Smith, Crombag, & Koya, 2018). Furthermore, a key follow‐up question is whether unique gene alterations in Fos‐positive neurons play causal roles in mediating drug seeking, which can be answered in future studies by manipulating the molecular composition of Fos‐expressing neurons using transgenic animals (Cruz et al., 2013).

Lastly, our data here do not refute previous functional studies on the specific roles of DMS and AIT in incubation of Meth craving after forced abstinence (Li, Carreria, et al., 2018; Li, Rubio, et al., 2015), because inactivation of DMS or AIT has no effect on ongoing food self‐administration. Caprioli et al. (Caprioli et al., 2017) further demonstrate the specific role of DMS in Meth seeking by showing that ablation of DMS neurons activated by food seeking has no effect of Meth‐seeking behaviors after voluntary abstinence.

## 5 CONCLUDING REMARKS

Our FACS study demonstrated that DMS and AIT Fos‐positive neurons, activated either during the relapse test or by novel context, exhibit distinct molecular profiles (compared with their respective Fos‐negative neurons), which reveals region specificity of molecular profiles of behaviorally activated neurons. Unexpectedly, gene alterations of candidate genes in both brain regions were similar in rats with a history of saline or Meth self‐administration. This finding underscores the importance of incorporating nondrug control groups in molecular studies of behaviorally activated neurons in future studies.

## CONFLICT OF INTEREST

The authors declare that they do not have any conflicts of interest (financial or otherwise) related to the text of the paper.

## AUTHOR CONTRIBUTIONS

XL designed and performed the studies, and drafted the manuscript. ID and OML performed the studies and analyzed the data. JZ and RC performed the FACS studies. FJR drafted the manuscript.

## Data Availability

The data that support the findings of this study are available from the corresponding author upon reasonable request.
