# Supplementary material for: Distinct gene alterations between Fos‐expressing striatal and thalamic neurons after withdrawal from methamphetamine self‐administration
Source: Brain Behav. 2019 Jul 31;9(9):e01378. doi: 10.1002/brb3.1378 (PMC6749486; doi:10.1002/brb3.1378)
Supplement: Supplementary file 1 [file BRB3-9-e01378-s001.docx]

**Table S1.** Primer/probe sequences for FACS-isolated neurons

| Gene | TaqMan probe or primer/probe | Forward primer | Reverse primer |
| --- | --- | --- | --- |
| *Pde10a* | TCCCATCGAGACCGC | CGCTGAACCTCCACAACCA | CGCAGGCAGTCATCATCAAG |
| *Neun* | CACTCCAACAGCGTGAC | GGCCCCTGGCAGAAAGTAG | TTCCCCCTGGTCCTTCTGA |
| *Gapdh* | CTCATGACCACAGTCCA | GACAACTTTGGCATCGTGGAA | CACAGTCTTCTGAGTGGCAGTGA |
| *Actb* | ATGAAGATCAAGATCATTGCT | AGAAGGAGATTACTGCCCTG | CCACCAATCCACACAGAGTACTT |
| *c-fos* | Rn00487426_g1* |  |  |
| *Erg1* | Rn00561138_m1* |  |  |
| *Arc* | Rn00571208_g1* |  |  |
| *Npas4* | Rn01454622_g1 |  |  |
| *Fosb* | CGAGAAGAAACACTTACC | CCAGAGCCAGGCCTAGAAGA | CTGCGAACCCTTCGTTTTTC |
| *Bdnf* | TGGTCAGTGGCTGGC | GGAGACCCTCCGCAACTGT | GAGCTATGATGTATCTTAGTGGGTATGAG |
| *Trkb1* | CATGAAAGGCCCAGCTT | TGGCGAGACATTCCAAGTTTG | AGAGTCATCGTCGTTGCTGATG |
| *Gria1* | Rn00709588_m1* |  |  |
| *Gria2* | Rn00568514_m1* |  |  |
| *Gria3* | Rn00583547_m1* |  |  |
| *Grin1* | Rn01436038_m1* |  |  |
| *Grin2a* | Rn00561341_m1* |  |  |
| *Grin2b* | Rn00680474_m1* |  |  |
| *Grm1* | Rn01440619_m1* |  |  |
| *Grm5* | Rn00690337_m1* |  |  |
| *Hdac1* | Rn01519308_g1* |  |  |
| *Hdac2* | Rn01193634_g1* |  |  |
| *Hdac3* | Rn00584926_m1* |  |  |
| *Hdac4* | Rn01427040_m1* |  |  |
| *Hdac5* | Rn01464245_m1* |  |  |
| *Sirt1* | CAGTGTCATGGTTCCTT | TTGCAGGAATCCAAAGGATCA | CAAATCAGGCAAGATGCTGTTG |
| *Sirt2* | Rn01457502_m1* |  |  |
| *Crebbp* | Rn01427040_m1* |  |  |
| *Suv39h1* | Rn01528294_g1* |  |  |
| *Ehmt1 (GLP)* | Rn01435512_m1* |  |  |
| *Ehmt2 (G9a)* | Rn0152918_m1* |  |  |
| *Kdm1a* | Rn01181029_m1* |  |  |
| *Kmt2a*  *(Mll1)* | CAAACAGACTGACCAGCC | GCCCAGCTCTGCAAGATAGAGA | ATCACTTTCTTGACCCTGTGCTTT |
| *Dnmt3a* | Rn01027162_g1* |  |  |
