# Supplementary material for: Distinct gene alterations between Fos‐expressing striatal and thalamic neurons after withdrawal from methamphetamine self‐administration
Source: Brain Behav. 2019 Jul 31;9(9):e01378. doi: 10.1002/brb3.1378 (PMC6749486; doi:10.1002/brb3.1378)
Supplement: Supplementary file 2 [file BRB3-9-e01378-s002.docx]

**Table S2. Behavioral and qPCR statistics**

| **Figure number** | **Factor name** | **F-value or t-value** | **p-value** | **Partial Eta^2^** |
| --- | --- | --- | --- | --- |
| Fig. 1c Training | Infusions:  Drug (between)  Training day (within) (Greenhouse)  Drug * Training day    Lever Presses:  Drug (between)  Lever (within)  Training day (within)  Lever * Drug  Lever * Training day  Drug * Training day  Lever * Training day * Drug | F_1,22_=34.3  F_3,72_=3.6  F_3,72_=37.3      F_1,22_=3.9  F_1,22_=278.1  F_2,57_=6.6  F_1,22_=115.1  F_9,79_=5.0  F_2,57_=14.0  F_3,79_=8.8 | <0.001*  0.013*  <0.001*      0.061  0.001*  0.001*  <0.001*  0.002*  <0.001*  <0.001* | 0.609  0.143  0.629      0.151  0.927  0.231  0.840  0.186  0.388  0.286 |
| Fig. 1d Relapse Test | Total Responses:  Lever (Within)  Drug (between)  Lever*Drug  Time Course:  Time Course (Within)  Drug (Between)  Time Course * Drug  Time Course:  30 min 60 min 90 min 120 min | F_1,22_=77.2  F_1,22_= 32.0  F_1,22_=36.5  F_1.7,36.9_=43.0  F_1,22_=35.2  F_1.7,36.89_=12.7  t_22_=5.5  t_22_=4.1  t_22_=6.3  t_22_=5.0 | <.001*  <.001*  <.001*  <.0001*  <.0001*  <.0001*  <.0001*  <.0001*  <.0001*  <.0001* | 0.778  0.593  0.624  0.662  0.615  0.366 |
| Fig. 1e Fos count/percentage | Fos %:  DMS (between)  AIT (between)  Fos Count:  DMS (between)  AIT (between) | t_22_=0.08  t_22_=0.42  t_22_=0.54  t_22_=1.34 | 0.941  0.682  0.598  0.178 |  |
| Fig. 1f Fos mRNA | DMS  Fos-labeling (Within)  Drug (Between)  Fos-labeling*Drug  AIT  Fos-labeling  Drug  Fos-labeling*Drug | F_1,19_=40.9  F_1,19_=0.26  F_1,19_=0.54  F_1,19_=6.6  F_1,19_=1.6  F_1,19_=1.34 | <.001*  0.619  0.471  0.019*  0.216  0.253 | 0.683  0.013  0.028  0.257  0.079  0.068 |
| Fig 2a. DMS Gene Expression | Immediate early genes   - *Arc*   - Fos-labeling   - Drug   - Fos-labeling*Drug - *Egr1*   - Fos-labeling   - Drug   - Fos-labeling*Drug - *Npas4*   - Fos-labeling   - Drug   - Fos-labeling*Drug - *Fosb*   - Fos-labeling   - Drug   - Cell*Drug   *Bdnf* and *Trkb*   - *Bdnf*   - Fos-labeling   - Drug   - Fos-labeling*Drug - *Trkb*   - Fos-labeling   - Drug   - Fos-labeling*Drug   Glutamate receptors   - *Gria1*   - Fos-labeling   - Drug   - Fos-labeling*Drug - *Gria2*   - Fos-labeling   - Drug   - Cell*Drug - *Gria3*   - Fos-labeling   - Drug   - Fos-labeling*Drug - *Grin1*   - Fos-labeling   - Drug   - Fos-labeling*Drug - *Grin2a*   - Fos-labeling   - Drug   - Fos-labeling*Drug - *Grin2b*   - Fos-labeling   - Drug   - Fos-labeling*Drug - *Grm1*   - Fos-labeling   - Drug   - Fos-labeling*Drug - *Grm5*   - Fos-labeling   - Drug   - Fos-labeling*Drug   Epigenetic enzymes   - *Hdac1*   - Fos-labeling   - Drug   - Fos-labeling*Drug - *Hdac2*   - Fos-labeling   - Drug   - Fos-labeling*Drug - *Hdac3*   - Fos-labeling   - Drug   - Fos-labeling*Drug - *Hdac4*   - Fos-labeling   - Drug   - Fos-labeling*Drug - *Hdac5*   - Fos-labeling   - Drug   - Fos-labeling*Drug - *Sirt1*   - Fos-labeling   - Drug   - Fos-labeling*Drug - Sirt2   - Fos-labeling   - Drug   - Fos-labeling*Drug - *Crebbp*   - Fos-labeling   - Drug   - Fos-labeling*Drug - *Suv39h1*   - Fos-labeling   - Drug   - Fos-labeling*Drug - *G9a*   - Fos-labeling   - Drug   - Fos-labeling*Drug - *Glp*   - Fos-labeling   - Drug   - Fos-labeling*Drug - *Kdm1a*   - Fos-labeling   - Drug   - Fos-labeling*Drug - *Mll1*   - Fos-labeling   - Drug   - Fos-labeling*Drug - *Dnmt3a*   - Fos-labeling   - Drug   - Fos-labeling*Drug | F_1,20_=28.7  F_1,20_=0.02  F_1,20_=0.12  F_1,22_=38.1  F_1,22_=0.16  F_1,22_=0.57  F_1,21_=44.1  F_1,21_=0.44  F_1,21_=0.74  F_1,20_=34.2  F_1,20_=1.2  F_1,20_=1.2  F_1,17_=0.95  F_1,17_= 2.5  F_1,17_=0.09  F_1,22_= 40.6  F_1,22_=1.2  F_1,22_=0.17  F_1,20_=0.14  F_1,20_=0.20  F_1,20_=0.13  F_1,22_=1.8  F_1,22_=0.008  F_1,22_=0.83  F_1,22_=16.34  F_1,22_=3.3  F_1,22_=0.34  F_1,18_=1209  F_1,18_=1.6  F_1,18_=0.59  F_1,19_=4.78  F_1,19_=3.5  F_1,19_=0.04  F_1,21_=20.1  F_1,21_=0.08  F_1,21_=1.1  F_1,20_=17.7  F_1,20_=0.70  F_1,20_=0.06  F_1,20_=7.8  F_1,20_=0.10  F_1,20_=0.19  F_1,19_=0.09  F_1,19_=0.52  F_1,19_=0.10  F_1,20_=5.5  F_1,20_=6.6  F_1,20_=1.7  F_1,21_=21.1  F_1,21_=0.01  F_1,21_=0.67  F_1,18_=2.1  F_1,18_=4.1  F_1,18_=3.8  F_1,19_=21.2  F_1,19_=2.1  F_1,19_=0.68  F_1,19_=6.7  F_1,19_=0.11  F_1,19_=1.4  F_1,19_=3.8  F_1,19_=0.14  F_1,19_=5.8  F_1,20_=10.6  F_1,20_=0.60  F_1,20_=0.66  F_1,17_=5.8  F_1,17_=0.12  F_1,17_=0.70  F_1,21_=0.02  F_1,21_=0.003  F_1,21_=1.1  F_1,18_=7.1  F_1,18_=0.17  F_1,18_=3.3  F_1,20_=9.4  F_1,20_=2.9  F_1,20_=0.59  F_1,22_=2.0  F_1,22_=0.49  F_1,22_=0.26  F_1,19_=8.2  F_1,19_=1.5  F_1,19_=0.02 | <0.001*  0.880  0.732  <0.001*  0.694  0.460  <0.001*  0.514  0.398  <0.001*  0.284  0.282  0.342  0.134  0.773  <0.001*  0.277  0.683  0.713  0.659  0.727  0.191  0.930  0.371  0.001*  0.083  0.566  0.003*  0.220  0.451  0.042  0.077  0.838  <0.001*  0.784  0.314  <0.001*  0.413  0.805  0.011  0.757  0.671  0.772  0.480  0.750  0.029  0.018  0.213  <0.001*  0.759  0.421  0.169  0.059  0.066  <.001*  0.167  0.419  0.018  0.746  0.249  0.068  0.712  0.027  0.004*  0.448  0.427  0.028  0.731  0.416  0.884  0.954  0.303  0.016  0.684  0.086  0.006  0.103  0.451  0.170  0.491  0.616  0.010  0.232  0.888 | 0.589  0.001  0.006  0.634  0.007  0.025  0.678  0.021  0.034  0.631  0.057  0.058  0.053  0.127  0.005  0.649  0.053  0.008  0.007  0.010  0.006  0.076  0.000  0.036  0.427  0.131  0.015  0.400  0.082  0.032  0.201  0.156  0.002  0.489  0.004  0.048  0.469  0.034  0.003  0.281  0.005  0.009  0.005  0.027  0.005  0.217  0.249  0.076  0.501  0.005  0.031  0.102  0.185  0.176  0.527  0.098  0.035  0.260  0.006  0.069  0.165  0.007  0.233  0.347  0.029  0.032  0.253  0.007  0.039  0.001  0.000  0.051  0.282  0.009  0.155  0.319  0.127  0.029  0.084  0.022  0.012  0.302  0.074  0.001 |
| Fig 2b. AIT Gene Expression | Immediate early genes   - *Arc*   - Fos-labeling   - Drug   - Fos-labeling*Drug - *Egr1*   - Fos-labeling   - Drug   - Fos-labeling*Drug - *Npas4*   - Fos-labeling   - Drug   - Fos-labeling*Drug - *Fosb*   - Fos-labeling   - Drug   - Fos-labeling*Drug   *Bdnf* and *Trkb*   - *Bdnf*   - Fos-labeling   - Drug   - Fos-labeling*Drug - *Trkb*   - Fos-labeling   - Drug   - Fos-labeling*Drug   Glutamate Receptors   - *Gria1*   - Fos-labeling   - Drug   - Fos-labeling*Drug - *Gria2*   - Fos-labeling   - Drug   - Fos-labeling*Drug - *Gria3*   - Fos-labeling   - Drug   - Fos-labeling*Drug - *Grin1*   - Fos-labeling   - Drug   - Fos-labeling*Drug - *Grin2a*   - Fos-labeling   - Drug   - Fos-labeling*Drug - *Grin2b*   - Fos-labeling   - Drug   - Fos-labeling*Drug - *Grm1*   - Fos-labeling   - Drug   - Fos-labeling*Drug - *Grm5*   - Fos-labeling   - Drug   - Fos-labeling*Drug   Epigenetic Enzymes   - *Hdac1*   - Fos-labeling   - Drug   - Fos-labeling*Drug - *Hdac2*   - Fos-labeling   - Drug   - Fos-labeling*Drug - *Hdac3*   - Fos-labeling   - Drug   - Fos-labeling*Drug - *Hdac4*   - Fos-labeling   - Drug   - Fos-labeling*Drug - *Hdac5*   - Fos-labeling   - Drug   - Fos-labeling*Drug - *Sirt1*   - Fos-labeling   - Drug   - Fos-labeling*Drug - *Sirt2*   - Fos-labeling   - Drug   - Fos-labeling*Drug - Crebbp   - Fos-labeling   - Drug   - Fos-labeling*Drug - Suv39h1   - Fos-labeling   - Drug   - Fos-labeling*Drug - *G9a*   - Fos-labeling   - Drug   - Fos-labeling*Drug - *Glp*   - Fos-labeling   - Drug   - Fos-labeling*Drug - *Kdm1a*   - Fos-labeling   - Drug   - Fos-labeling*Drug - *Mll1*   - Fos-labeling   - Drug   - Fos-labeling*Drug - *Dnmt3a*   - Fos-labeling   - Drug   - Fos-labeling*Drug | F_1,15_=0.03  F_1,15_=0.99  F_1,15_=0.22  F_1,15_=18.2  F_1,15_=0.02  F_1,15_=0.26  F_1,20_=5.4  F_1,20_=0.11  F_1,20_=2.23  F_1,13_=19.0  F_1,13_=0.71  F_1,13_=0.32  F_1,18­_=3.6  F_1,18_=3.4  F_1,18_=1.2  F_1,21_=13.0  F_1,21_=0.01  F_1,21_=0.19  F_1,20_=0.01 F_1,20_=0.23  F_1,20_=0.002  F_1,21_=1.0  F_1,21_=0.08  F_1,21_=0.35  F_1,21_=1.1  F_1,21_=0.14  F_1,21_=0.82  F_1,20_=15.4  F_1,20_=0.14  F_1,20_=1.3  F_1,19_=6.8  F_1,19_=0.18  F_1,19_=0.09  F_1,20_=0.77  F_1,20_=1.0  F_1,20_=0.47  F_1,21_=3.0  F_1,21_=0.22  F_1,21_=0.60  F_1,17_=0.03  F_1,17_=0.08  F_1,17_=0.80  F_1,19_=0.02  F_1,19_=1.7  F_1,19_=0.26  F_1,19_=0.17  F_1,19_=0.22  F_1,19_=0.94  F_1,21_=1.1  F_1,21_=0.62  F_1,21_=0.001  F_1,17_=6.4  F_1,17_=0.21  F_1,17_=3.01  F_1,21_=14.0  F_1,21_=0.01  F_1,21_=0.03  F_1,17_=1.9  F_1,17_=0.02  F_1,17_=0.40  F_1,17_=10.2  F_1,17_=0.15  F_1,17_=1.3  F_1,19_=1.6  F_1,19_=0.07  F_1,19_=0.04  F_1,16_=0.00  F_1,16_=1.6  F_1,16_=0.003  F_1,20_=0.24  F_1,20_=0.72  F_1,20_=0.003  F_1,21_=2.3  F_1,21_=0.00  F_1,21_=0.97  F_1,19_=0.07  F_1,19_=0.08  F_1,19_=0.06  F_1,20_=3.3  F_1,20_=0.45  F_1,20_=0.68  F_1,19_=6.1  F_1,19_=1.3  F_1,19_=1.7 | 0.859  0.337  0.643  0.001*  0.884  0.619  0.031  0.742  0.148  0.001*  0.416  0.584  0.073  0.082  0.298  0.002*  0.909  0.668  0.912  0.636  0.963  0.323  0.784  0.559  0.304  0.712  0.375  0.001*  0.713  0.281  0.018  0.672  0.764  0.395  0.327  0.501  0.099  0.645  0.447  0.863  0.783  0.384  0.898  0.204  0.613  0.681  0.644  0.344  0.30  0.44  0.97  0.022  0.652  0.101  0.001*  0.92  0.871  0.182  0.903  0.534  0.005  0.705  0.270  0.217  0.792  0.842  0.986  0.228  0.960  0.629  0.406  0.960  0.147  0.995  0.336  0.798  0.780  0.838  0.083  0.511  0.420  0.023  0.277  0.209 | 0.002  0.062  0.015  0.549  0.001  0.017  0.213  0.006  0.102  0.594  0.052  0.024  0.167  0.159  0.060  0.382  0.001  0.009  0.001  0.011  0.000  0.046  0.004  0.016  0.050  0.007  0.038  0.434  0.007  0.058  0.263  0.010  0.005  0.036  0.048  0.023  0.124  0.010  0.028  0.002  0.005  0.045  0.001  0.083  0.014  0.009  0.011  0.047  0.050  0.029  0.000  0.272  0.012  0.151  0.40  0.000  0.001  0.102  0.001  0.023  0.375  0.009  0.072  0.079  0.004  0.002  0.000  0.089  0.000  0.012  0.035  0.000  0.097  0.000  0.044  0.004  0.004  0.002  0.143  0.022  0.033  0.243  0.062  0.082 |
| Fig. 3D Fos mRNA | AIT  DMS | t_5_= -4.5  t_4_= -3.2 | 0.006*  0.032* |  |
| Figure 4. Novel Context DMS | *Arc*  *Egr1*  *Npas4*  *Fosb*  *Bdnf*  *Trkb*  *Gria1*  *Gria2*  *Gria3*  *Grin2a*  *Grin2b*  *Grm1*  *Grm5*  *Hdac1*  *Hdac2*  *Hdac3*  *Hdac4*  *Hdac5*  *Sirt1*  *Sirt2*  *Crebbp*  *Suv39h1*  *G9a*  *Glp*  *Kdm1a*  *Mll1*  *Dnmt3a* | t_6_=1.9  t_5_=1.1  t_5_=3.0  t_3_=2.1  t_5_=2.5  t_6_=0.35  t_6_=4.0  t_6_=1.2  t_6_=1.3  t_6_=1.9  t_5_=0.54  t_6_=2.9  t_6_=1.4  t_5_=0.20  t_6_=2.3  t_7_=0.78  t_5_=0.92  t_6_=0.45  t_6_=0.59  t_5_=0.49  t_6_=0.27  t_4_=1.62  t_6_=0.09  t_7_=0.14  t_6_=0.82  t_6_=1.6  t_4_=2.4 | 0.108  0.330  0.029  0.127  0.054  0.738  0.007  0.250  0.229  0.111  0.611  0.029  0.222  0.849  0.058  0.461  0.401  0.664  0.579  0.645  0.800  0.181  0.930  0.893  0.443  0.172  0.078 |  |
| Figure 4. Novel Context AIT | *Arc*  *Egr1*  *Npas4*  *Fosb*  *Bdnf*  *Trkb*  *Gria1*  *Gria2*  *Gria3*  *Grin2a*  *Grin2b*  *Grm1*  *Grm5*  *Hdac1*  *Hdac2*  *Hdac3*  *Hdac4*  *Hdac5*  *Sirt1*  *Sirt2*  *Crebbp*  *Suv39h1*  *G9a*  *Glp*  *Kdm1a*  *Mll1*  *Dnmt3a* | t_7_= 2.1  t_6_= 1.6  t_6_= 3.4  t_6_= 5.2  t_6_= 3.2  t_6_= 5.0  t_5_= 0.25  t_7_= 0.80  t_5_= 2.5  t_6_= 2.5  t_7_= 1.5  t_6_= 1.2  t_6_= 0.001  t_6_= 2.7  t_6_= 0.98  t_7_= 0.48  t_6_= 1.4  t_6_= 2.3  t_6_= 2.3  t_6_= 0.80  t_6_= 0.96  t_4_= 0.28  t_7_= 1.9  t_6_= 0.67  t_7_= 1.2  t_6_= 0.49  t_6_=0.08 | 0.071  0.154  0.015  0.002*  0.019  0.003*  0.811  0.446  0.057  0.045  0.189  0.284  0.992  0.036  0.366  0.643  0.209  0.064  0.065  0.453  0.376  0.796  0.105  0.525  0.280  0.637  0.938 |  |
