# Supplementary material for: Distinct gene alterations between Fos‐expressing striatal and thalamic neurons after withdrawal from methamphetamine self‐administration
Source: Brain Behav. 2019 Jul 31;9(9):e01378. doi: 10.1002/brb3.1378 (PMC6749486; doi:10.1002/brb3.1378)
Supplement: Supplementary file 3 [file BRB3-9-e01378-s003.docx]

**Table S3. Post-hoc tests in Fig. 2**

| **Figure number** | **Test** | **t value** | **p value** |
| --- | --- | --- | --- |
| Fig2A. DMS Gene Expression | IEG’s 🡪 Paired Samples t-test   - *Arc*   - Saline   - Meth - *Egr1*   - Saline   - Meth - *Npas4*   - Saline   - Meth - *Fosb*   - Saline   - Meth   *Bdnf* and *Trkb*🡪 Paired Samples t-test   - *Trkb*   - Saline   - Meth   Glutamate Receptors 🡪 Paired Samples t-test   - *Gria3*   - Saline   - Meth - *Grin1*   - Saline   - Meth - *Grin2b*   - Saline   - Meth - *Grm1*   - Saline   - Meth   Epigenetic Enzymes   - *Hdac3*   - Saline   - Meth - *Hdac5*   - Saline   - Meth - *Crebbp*   - Saline   - Meth | t_9_= -4.3  t_11_= -3.5  t_9_= -3.7  t_13_= -5.1  t_8_= -4.5  t_13_= -4.9  t_8_= -3.6  t_12_= -5.0  t_9_= -4.0  t_13_= -5.1  t_9_= -2.6  t_13_= -3.3  t_8_= -3.5  t_10_= -1.8  t_9_= -3.6  t_12_= -2.6  t_9_= -2.8  t_11_= -3.2  t_9_= -2.8  t_-12_= -4.1  t_7_= -3.6  t_12_= -3.8  t_7_= -2.6  t_13_= -2.0 | 0.003*  0.005*  0.0046*  0.000*  0.002*  0.000*  0.007  0.000*  0.003*  0.000*  0.027  0.006  0.009  0.104  0.0054  0.022  0.021  0.008  0.021  0.002*  0.009  0.002*  0.034  0.067 |
| Fig2B. AIT Gene Expression | IEG’s 🡪 Paired Sample t test   - *Egr1*   - Saline   - Meth - *Fosb*   - Saline   - Meth   *Bdnf* and *Trkb*   - *Trkb*   - Saline   - Meth   Glutamate Receptors   - *Grin1*   - Saline   - Meth   Epigenetic Receptors   - *Hdac5*   - Saline   - Meth | t_7_= -2.7  t_8_= -3.6  t_4_= -3.2  t_9_= -3.9  t_9_= -2.8  t_13_= -2.6  t_8_= -2.7  t_12_=-3.4  t_9_=-2.3  t_12_=-3.1 | 0.031  0.007  0.033  0.004*  0.022  0.024  0.026  0.006  0.05  0.009 |
